# Loneliness and Isolation as Barriers to Mental Health Services in a Rural Community

**DOI:** 10.1093/geroni/igab046.3704

**Published:** 2021-12-17

**Authors:** Robert Maiden, Danielle Gagne, Bert Hayslip

**Affiliations:** 1 Alfred University, Alfred, New York, United States; 3 University of North Texas at Denton, Denton, Texas, United States

## Abstract

As America grapples with COVID-19, issues regarding mental health have been of rising concern, particularly among those who are isolated. According to the May 2021 American Perspectives Survey, “Americans report having fewer close friendships than they once did, talking to their friends less often, and relying less on their friends for personal support1.” Additionally, 49% have three or fewer close friends in 2021, compared to 27% in 1990. 17% have no friends in their core social network. Loneliness has been associated with physical and mental health risks. We sought to explore whether loneliness was also a barrier to seeking mental health services. 90 surveys were collected from rural New York. Respondents were aged 51 to 90, Caucasian (96.6%), and female (73.3% vs, 26.7%). Overall, 34.8% said they lived alone. 29.2% would seek mental health services for feelings of loneliness, while 75.4% would do so if isolated from family. Those who felt detached or isolated from others were significantly less likely to seek help from a counselor (r = - 0.25) or MD (r = - 0.37). Isolation also negatively related to measures on the resiliency scale. **Purpose**: - 0.22, Perseverance: - 0.33, being ok alone: - 0.32), and positively related to depression (r = .65). Those scoring higher on the “okay with being alone” scale had an increased likelihood of seeking counseling (r = 0.22). Thus, isolation and loneliness are complex topics. Intervention ought to be based on perceptions of being alone. Further research is needed.

